# Imaging of the brain–heart axis: prognostic value in a European setting

**DOI:** 10.1093/eurheartj/ehae162

**Published:** 2024-04-10

**Authors:** Nidaa Mikail, Dominik F Sager, Pimrapat Gebert, Achi Haider, Atanas Todorov, Susan Bengs, Noemi Sablonier, Isabelle Glarner, Adriana Vinzens, Nastaran Sang Bastian, Gioia Epprecht, Claudia Sütsch, Alessia Delcò, Michael Fiechter, Angela Portmann, Valerie Treyer, Susanne Wegener, Christoph Gräni, Aju Pazhenkottil, Caroline E Gebhard, Vera Regitz-Zagrosek, Felix C Tanner, Philipp A Kaufmann, Ronny R Buechel, Alexia Rossi, Catherine Gebhard

**Affiliations:** Department of Nuclear Medicine, University Hospital Zurich, Raemistrasse 100, 8091 Zurich, Switzerland; Center for Molecular Cardiology, University of Zurich, Wagistrasse 12, 8952, Schlieren, Switzerland; Department of Nuclear Medicine, University Hospital Zurich, Raemistrasse 100, 8091 Zurich, Switzerland; Center for Molecular Cardiology, University of Zurich, Wagistrasse 12, 8952, Schlieren, Switzerland; Department of Nuclear Medicine, University Hospital Zurich, Raemistrasse 100, 8091 Zurich, Switzerland; Center for Molecular Cardiology, University of Zurich, Wagistrasse 12, 8952, Schlieren, Switzerland; Institute of Biometry and Clinical Epidemiology, Charité—Universitätsmedizin Berlin, Berlin, Germany; Department of Nuclear Medicine, University Hospital Zurich, Raemistrasse 100, 8091 Zurich, Switzerland; Center for Molecular Cardiology, University of Zurich, Wagistrasse 12, 8952, Schlieren, Switzerland; Department of Radiology, Division of Nuclear Medicine and Molecular Imaging, Massachusetts General Hospital and Harvard Medical School, Boston, USA; Department of Nuclear Medicine, University Hospital Zurich, Raemistrasse 100, 8091 Zurich, Switzerland; Center for Molecular Cardiology, University of Zurich, Wagistrasse 12, 8952, Schlieren, Switzerland; Department of Nuclear Medicine, University Hospital Zurich, Raemistrasse 100, 8091 Zurich, Switzerland; Center for Molecular Cardiology, University of Zurich, Wagistrasse 12, 8952, Schlieren, Switzerland; Department of Nuclear Medicine, University Hospital Zurich, Raemistrasse 100, 8091 Zurich, Switzerland; Center for Molecular Cardiology, University of Zurich, Wagistrasse 12, 8952, Schlieren, Switzerland; Department of Nuclear Medicine, University Hospital Zurich, Raemistrasse 100, 8091 Zurich, Switzerland; Center for Molecular Cardiology, University of Zurich, Wagistrasse 12, 8952, Schlieren, Switzerland; Department of Nuclear Medicine, University Hospital Zurich, Raemistrasse 100, 8091 Zurich, Switzerland; Center for Molecular Cardiology, University of Zurich, Wagistrasse 12, 8952, Schlieren, Switzerland; Department of Nuclear Medicine, University Hospital Zurich, Raemistrasse 100, 8091 Zurich, Switzerland; Center for Molecular Cardiology, University of Zurich, Wagistrasse 12, 8952, Schlieren, Switzerland; Department of Nuclear Medicine, University Hospital Zurich, Raemistrasse 100, 8091 Zurich, Switzerland; Center for Molecular Cardiology, University of Zurich, Wagistrasse 12, 8952, Schlieren, Switzerland; Department of Nuclear Medicine, University Hospital Zurich, Raemistrasse 100, 8091 Zurich, Switzerland; Center for Molecular Cardiology, University of Zurich, Wagistrasse 12, 8952, Schlieren, Switzerland; Department of Nuclear Medicine, University Hospital Zurich, Raemistrasse 100, 8091 Zurich, Switzerland; Center for Molecular Cardiology, University of Zurich, Wagistrasse 12, 8952, Schlieren, Switzerland; Department of Nuclear Medicine, University Hospital Zurich, Raemistrasse 100, 8091 Zurich, Switzerland; Center for Molecular Cardiology, University of Zurich, Wagistrasse 12, 8952, Schlieren, Switzerland; Swiss Paraplegic Center, Nottwil, Switzerland; Department of Nuclear Medicine, University Hospital Zurich, Raemistrasse 100, 8091 Zurich, Switzerland; Center for Molecular Cardiology, University of Zurich, Wagistrasse 12, 8952, Schlieren, Switzerland; Department of Nuclear Medicine, University Hospital Zurich, Raemistrasse 100, 8091 Zurich, Switzerland; Department of Neurology and Clinical Neuroscience Center, University Hospital Zurich and University of Zurich, Zurich, Switzerland; Department of Cardiology, Inselspital, Bern University Hospital, University of Bern, Freiburgstrasse 20, 3010, Bern, Switzerland; Department of Nuclear Medicine, University Hospital Zurich, Raemistrasse 100, 8091 Zurich, Switzerland; Intensive Care Unit, Department of Acute Medicine, University Hospital Basel, University of Basel, Basel, Switzerland; University of Zurich, Zurich, Switzerland; Institute of Gender in Medicine (GiM), Charité—Universitätsmedizin Berlin, Berlin, Germany; Department of Cardiology, University Hospital Zurich, University of Zurich, Zurich, Switzerland; Department of Nuclear Medicine, University Hospital Zurich, Raemistrasse 100, 8091 Zurich, Switzerland; Department of Nuclear Medicine, University Hospital Zurich, Raemistrasse 100, 8091 Zurich, Switzerland; Department of Nuclear Medicine, University Hospital Zurich, Raemistrasse 100, 8091 Zurich, Switzerland; Center for Molecular Cardiology, University of Zurich, Wagistrasse 12, 8952, Schlieren, Switzerland; Department of Nuclear Medicine, University Hospital Zurich, Raemistrasse 100, 8091 Zurich, Switzerland; Center for Molecular Cardiology, University of Zurich, Wagistrasse 12, 8952, Schlieren, Switzerland; Department of Cardiology, Inselspital, Bern University Hospital, University of Bern, Freiburgstrasse 20, 3010, Bern, Switzerland

**Keywords:** Psychological stress, Stress-related neural activity, Amygdala, Ventromedial prefrontal cortex, Haematopoietic tissue activity, MACE, Mortality, ^18^F-FDG-PET/CT

## Abstract

**Background and Aims:**

Increasing data suggest that stress-related neural activity (SNA) is associated with subsequent major adverse cardiovascular events (MACE) and may represent a therapeutic target. Current evidence is exclusively based on populations from the U.S. and Asia where limited information about cardiovascular disease risk was available. This study sought to investigate whether SNA imaging has clinical value in a well-characterized cohort of cardiovascular patients in Europe.

**Methods:**

In this single-centre study, a total of 963 patients (mean age 58.4 ± 16.1 years, 40.7% female) with known cardiovascular status, ranging from ‘at-risk’ to manifest disease, and without active cancer underwent 2-[^18^F]fluoro-2-deoxy-D-glucose positron emission tomography/computed tomography between 1 January 2005 and 31 August 2019. Stress-related neural activity was assessed with validated methods and relations between SNA and MACE (non-fatal stroke, non-fatal myocardial infarction, coronary revascularization, and cardiovascular death) or all-cause mortality by time-to-event analysis.

**Results:**

Over a maximum follow-up of 17 years, 118 individuals (12.3%) experienced MACE, and 270 (28.0%) died. In univariate analyses, SNA significantly correlated with an increased risk of MACE (sub-distribution hazard ratio 1.52, 95% CI 1.05–2.19; *P* = .026) or death (hazard ratio 2.49, 95% CI 1.96–3.17; *P* < .001). In multivariable analyses, the association between SNA imaging and MACE was lost when details of the cardiovascular status were added to the models. Conversely, the relationship between SNA imaging and all-cause mortality persisted after multivariable adjustments.

**Conclusions:**

In a European patient cohort where cardiovascular status is known, SNA imaging is a robust and independent predictor of all-cause mortality, but its prognostic value for MACE is less evident. Further studies should define specific patient populations that might profit from SNA imaging.

## Structured Graphical Abstract

**Structured Graphical Abstract:**
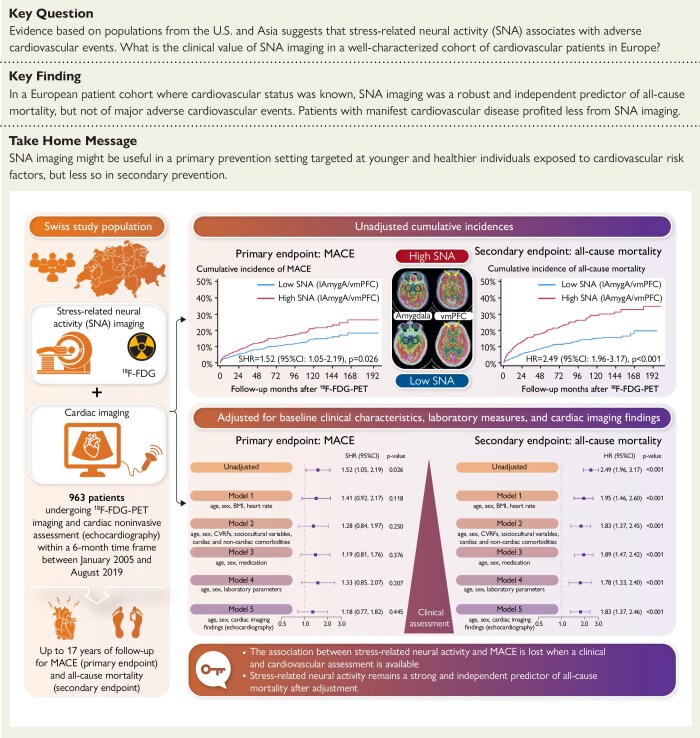
In a well-characterized European population (Switzerland) including patients with known comorbidities, SNA imaging is a predictor of MACE and all-cause mortality. However, after adjusting for baseline characteristics, the association between SNA imaging and MACE is lost, and SNA only remains a strong and independent predictor of all-cause mortality. ^18^F-FDG-PET, 2-[^18^F]fluoro-2-deoxy-D-glucose positron emission tomography; BMI, body mass index; CVRF, cardiovascular risk factor; HR, hazard ratio; lAmygA, left amygdala activity; MACE, major adverse cardiovascular events; SHR, sub-distribution hazard ratio; SNA, stress-related neural activity; vmPFC, ventromedial prefrontal cortex.

**See the editorial comment for this article ‘Unveiling the heart's silent whisperer: study of stress and the brain–heart connection in Europe’, by Z.A. Fayad and D. O'Connor, https://doi.org/10.1093/eurheartj/ehae193.**

## Introduction

Despite a thorough understanding of cardiovascular disease (CVD) pathophysiology and improved control of cardiovascular risk factors (CVRFs), CVDs remain the leading cause of death worldwide.^1^ Consequently, novel approaches aim to improve CVD risk stratification, notably research on the brain–heart axis.^2^ Indeed, the brain and the heart are connected through numerous neurological, hormonal, and immune pathways,^2–4^ Recent studies emphasized the role of the amygdalae, a region involved in processing stress responses, in predicting subsequent cardiovascular (CV) events,^5–7^ with amygdala metabolic activity (AmygA) serving as a surrogate for stress-related neural activity (SNA). 2-[^18^F]fluoro-2-deoxy-D-glucose positron emission tomography/computed tomography (^18^F-FDG-PET/CT) reliably quantifies AmygA and enables simultaneous estimation of arterial inflammation and haematopoietic tissue activity.

Despite the suitability of ^18^F-FDG-PET to investigate the brain–heart connection, its clinical value is unclear. Current evidence is exclusively based on U.S.^4,6–11^ and Asian^5,12–14^ populations, with limited information about CVD status and comorbidities. This constraint is significant given the numerous factors influencing SNA, especially in CV patients. Besides stress-related disorders,^15^ age,^16,17^ sex,^16^ obesity, diabetes, pre-existing atherosclerotic disease, or recent myocardial infarction are all linked with SNA.^14,17–20^ Moreover, socioeconomic and lifestyle variables, including education, alcohol consumption, and physical exercise, also influence SNA, suggesting that social disparities affect health through neurobiological mechanisms^6,8–10,14^. Medications, particularly statins or anti-inflammatory drugs, negatively correlate with SNA.^4,21^ Factors potentially affecting SNA increase with comorbidities, such as in patients with chronic inflammatory diseases or with heart failure (HF), with recent evidence linking reduced SNA and arrhythmic events risk.^4,12^

Given the many parameters influencing SNA, especially in the typical aged CV patient, this study sought to evaluate the incremental prognostic value of SNA imaging over a detailed patient history, baseline laboratory measurements, and cardiac imaging findings in a real-world European scenario.

## Methods

### Study design and population

**Figure 1:**
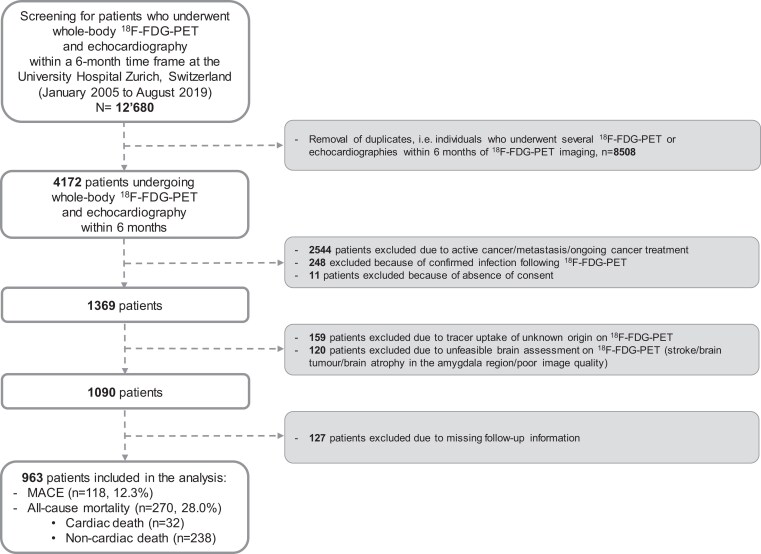
Flowchart depicting patient recruitment and exclusion. ^18^F-FDG-PET, 2-[^18^F]fluoro-2-deoxy-D-glucose positron emission tomography; MACE, major adverse cardiovascular events

Patients who underwent clinically indicated whole-body ^18^F-FDG-PET and echocardiography within a 6-month time frame at the University Hospital Zurich, Switzerland, between January 2005 and August 2019 were screened for inclusion in this single-centre, retrospective, longitudinal, observational imaging study that evaluated the relationship between SNA and subsequent major adverse cardiovascular events (MACE) or all-cause mortality (*Figure 1*). At the University Hospital Zurich, the standard acquisition field of view includes the brain, whether patients are addressed for cancer-, infection-, or inflammatory-related indications. The main indications for ^18^F-FDG-PET were cancer suspicion, follow-up of patients with cancer history, fever of unknown origin/suspected infection, or inflammatory disorders. The main indications for echocardiography were periodic control for known ischaemic or structural heart disease and repeat assessment in patients with cancer history and potentially cardiotoxic anticancer treatments. Amongst these patients, *n* = 4172 patients ≥ 18 years were selected. The pre-defined inclusion criteria at the time of ^18^F-FDG-PET imaging were (i) absence of cancer and/or remission from cancer for at least one year before imaging, (ii) absence of acute or chronic infection, (iii) absence of acute inflammatory or autoimmune disease, (iv) incomplete acquisitions precluding brain exploration, and (v) unstable medical conditions. Patients whose ^18^F-FDG-PET images could not be analysed due to insufficient image quality, patients who did not provide informed consent, and patients for whom follow-up information was missing (*n* = 127) were also excluded. The latter sample did not differ in terms of baseline characteristics (age, sex, comorbidities, and CVRFs) from patients included in the analysis. Patients were not selected based on their CVD status. To ensure adequate follow-up information for the study endpoints, patients, their next of kin, or their legal representative were contacted by phone. When the mortality status was uncertain, the Swiss National Death Register was consulted. Finally, 963 patients [392 (41%) women] were included in the study (*Figure 1*).

The study was approved by the Cantonal Ethics Committee Zurich (BASEC 2017–01112), and all patients included gave written informed consent.

### Data acquisition and definitions

Patient data were retrieved using hospital electronic medical records. Individual charts were reviewed to collect vital signs, CVRFs, presence and current status of CVD, medication, cardiac imaging findings derived from transthoracic echocardiography, laboratory parameters including routine inflammatory parameters, serum levels of N-terminal pro-brain natriuretic peptide (NT-proBNP), glucose, and renal function parameters, as well as socio-demographic data comprising civil status and professional skill level as per the International Standard Classification of Occupations.^22^ Collected data and their definitions are summarized in Supplementary data online, *Table S1*.

### Image acquisition and analysis

#### ^18^F-fluorodeoxyglucose positron emission tomography with computed tomography

^18^F-FDG-PET was performed on two PET/CT scanners (Discovery VCT or Discovery RX, GE Healthcare, Milwaukee, WI, USA) using standardized clinical protocols. Briefly, patients were asked to fast for ≥4 h before ^18^F-FDG injection. After measuring glucose level, ^18^F-FDG was administered via a peripheral vein at a dose of 1.3–1.8 MBq/kg based on body mass index (BMI) (minimal and maximal activities: 90 and 300 MBq, respectively), and the tracer uptake time was set to 45–60 min. A non-gated, non-contrast-enhanced CT scan (120 keV, ∼50 mAs) from the skull to mid-thigh was obtained for attenuation correction of PET images and anatomical localization of ^18^F-FDG uptake. Following CT acquisition, the corresponding PET images were acquired in 3D mode over 10 min scanning cranially to caudally. PET data were recalculated to provide images of standardized uptake values (SUV) based on total body weight and injected dose. Using a dedicated workstation (AW 5.0 GE Healthcare, Milwaukee, WI, USA) and software (PMOD software, version 4.003, PMOD Technologies Ltd) PET/CT images were fused, and a comprehensive assessment was performed to quantify ^18^F-FDG uptake in different tissues.

#### Measurement of stress-related neural metabolic activity

Stress-related neural activity was quantified using validated methods.^16,21,23,24^ One fully trained nuclear medicine specialist (N.M.) and one fully trained radiologist (A.R.) blinded to clinical information assessed ^18^F-FDG brain uptake. After cropping the whole-body PET scan for the skull, the brain gyri were segmented using the Maximum Probability Atlas in the NEURO tool of PMOD. Regions of interest (ROIs) corresponding to the right (rAmygA), left amygdala (lAmygA), and ventromedial prefrontal cortex (vmPFC, a counter-regulatory centre under stressful conditions^11^) were identified. ^18^F-FDG accumulation was measured as the mean standardized ^18^F-FDG uptake value (SUVmean) for each ROI. The primary measure for SNA (meanAmygA) was defined as the average of the SUVmean of bilateral AmygA. Given the influence of amygdala laterality in emotion processing,^25–27^ right and left AmygA were analysed separately [SNA (lAmygA) and SNA (rAmygA)]. To reduce inter-individual variability, AmygA must be corrected to a background brain region not involved in stress processing, i.e. the cerebellum or the temporal lobes.^9,10^ While this approach helps to standardize AmygA measurements, it does not account for the activation of other cerebral regions during mental stress, such as the prefrontal cortex.^28,29^ Recent studies point to an amygdala-vmPFC interplay for MACE prediction, with the vmPFC acting as a counter-player to the amygdala during psychological stress.^6,11^ As such, vmPFC activation during mental stress triggers the parasympathetic system and inhibits the sympathetic system.^30,31^ Given that the amygdala-to-vmPFC ratio might account for a potential cardioprotective effect of the vmPFC, not captured by the AmygA-to-background variable, AmygA (individually and averaged) was corrected for vmPFC activity (SUVmean) in our primary analysis (SNA [AmygA/vmPFC]).^11^ Still, to compare how different reference regions affect the association between AmygA and outcomes, we performed a supplementary analysis where AmygA was corrected for temporal lobe activity (AmygA/temp). We tested four parameters, namely, averaged bilateral SNA (meanAmygA/vmPFC), left SNA (lAmygA/vmPFC), left SNA (lAmygA/temp), and right SNA (rAmygA/vmPFC). More details are provided in Supplementary data online, *Figures S1* and *S2*. Details regarding the measurement of haematopoietic activity (HTA) in the bone marrow and spleen, echocardiography assessment, and laboratory parameters are provided in Supplementary Material.

#### Follow-up and study outcomes

Follow-up data were collected through telephone interviews with the patients, their next of kin, or their legal representative. Hospital records and clinical documents were screened for confirmation. The composite primary study outcome was MACE, including non-fatal stroke, non-fatal myocardial infarction, coronary revascularization, and CV death.^32^ Major adverse cardiovascular events adjudication was performed by one cardiologist (C.G.) and one cardiac imaging radiologist (A.R.), blinded to imaging data. The secondary endpoint, all-cause mortality, comprised CV and non-CV death. The last follow-up date was 1 October 2022, with censoring for patients not reaching the endpoints during the study period.

### Statistical approach

Details are provided in Supplementary Material. Briefly, to identify cut-points in SNA (lAmgyA/vmPFC and lAmygA/temp), we performed a classification and regression tree (CART) analysis for time-to-event data based on the all-cause mortality endpoint using the *cart* command in Stata MP/18.^33^ Bivariate and multivariable analyses were performed to investigate the association between SNA (AmgyA/vmPFC) [as continuous and as dichotomized variables (high vs. low based on CART analysis)] and time-to-event outcomes, where SNA (lAmgyA/vmPFC) had a linear effect on time-to-MACE and time-to-death. We performed Fine and Gray’s proportional sub-distribution hazards models for time-to-MACE, assuming that non-CV death was a competing risk, and we applied Cox regression models for all-cause mortality. To explore the impact of competing variables on the association between SNA (lAmgyA/vmPFC) and our study endpoint, we built five different models, defining the covariables *a priori*: we first evaluated the impact of basic demographic variables on the association between SNA (lAmgyA/vmPFC) and MACE/all-cause mortality (Model 1). Next, we assessed the effect of CVRFs, cardiac and non-cardiac comorbidities, as well as sociocultural variables on the association between SNA (lAmgyA/vmPFC) and MACE/all-cause mortality (Model 2), followed by medication *(*Model 3), laboratory parameters (Model 4), and cardiac imaging findings (Model 5). All statistical tests were performed using Stata MP/18 (StataCorp, 2023, College Station, TX, USA). To compare baseline characteristics between patients with high and low SNA (lAmgyA/vmPFC) or between those who experienced the study endpoints (MACE and all-cause mortality), we performed a χ^2^ test for categorical variables and an independent *t*-test of Mann–Whitney *U* test for continuous variables, depending on the distribution.

## Results

### Demographic and clinical characteristics of the study population

Overall, 963 individuals (mean age 58.4 ± 16.1 years, 41% female) met the inclusion criteria and were followed for a median of 5 years (IQR: 3–9 years, *Figure 1*). Amongst them, 340 (35.3%) patients had known CVD, including CAD (18.9%) and/or structural heart disease (22.3%). A total of 355 (36.9%) patients had a cancer history, while 34 (3.5%) patients had chronic inflammatory disease. The most common cancers were lymphoma (48.5%), melanoma (13.6%), head and neck cancer (13.1%), breast cancer (13%), and lung cancer (7%). Cardiovascular risk factor prevalence was high as indicated in *Table 1*.

**Table 1:** Patient’s baseline characteristics stratified by stress-related neural activity (lAmygA/vmPFC)

Patient’s characteristics	Total (*n* = 963)	SNA (lAmygA/vmPFC)		*P*-value
		Low^a^ (*n* = 656)	High^a^ (*n* = 307)	
Age (years)—mean (SD)	58.43 (16.07)	55.66 (16.53)	64.36 (13.24)	<.001
Sex				1.00
Male	571 (59.29%)	389 (59.30%)	182 (59.28%)	
Female	392 (40.71%)	267 (40.70%)	125 (40.72%)	
BMI (kg/m^2^)—mean (SD)	25.59 (5.10)	25.42 (5.03)	25.93 (5.23)	.18
Socioeconomic variables				
Living situation				.42
Partnership/married	553 (57.42%)	371 (56.55%)	182 (59.28%)	
Living alone	410 (42.58%)	285 (43.45%)	125 (40.72%)	
Occupation skill level				.28
Low	794 (87.54%)	536 (86.73%)	258 (89.27%)	
High	113 (12.46%)	82 (13.27%)	31 (10.73%)	
Comorbidities				
Comorbidities—non-cardiac	98 (10.18%)	56 (8.54%)	42 (13.68%)	.014
Comorbidities—cardiac	340 (35.31%)	207 (31.55%)	133 (43.32%)	<.001
Known CAD	182 (18.90%)	108 (16.46%)	74 (24.10%)	.005
Known structural or valvular heart disease, arrhythmia	215 (22.33%)	123 (18.75%)	92 (29.97%)	<.001
History of cancer	355 (36.86%)	264 (40.24%)	91 (29.64%)	.001
Chronic inflammatory disease	34 (3.53%)	21 (3.20%)	13 (4.23%)	.42
Cardiovascular risk factors				
Obesity	134 (16.54%)	81 (14.81%)	53 (20.15%)	.055
Diabetes	189 (19.65%)	108 (16.49%)	81 (26.38%)	<.001
Dyslipidaemia	289 (30.04%)	185 (28.24%)	104 (33.88%)	.076
Hypertension	446 (46.36%)	277 (42.29%)	169 (55.05%)	<.001
Family history of CAD	127 (13.20%)	88 (13.44%)	39 (12.70%)	.75
Smoking	492 (51.14%)	329 (50.23%)	163 (53.09%)	.41
Medication				
Blood pressure/heart failure medication	214 (22.22%)	121 (18.45%)	93 (30.29%)	<.001
Antiplatelet/anticoagulants	158 (16.41%)	89 (13.57%)	69 (22.48%)	<.001
Anti-inflammatory drugs	201 (20.87%)	119 (18.14%)	82 (26.71%)	.002
Antiarrhythmics	17 (1.77%)	8 (1.22%)	9 (2.93%)	.060
Antidepressants	55 (5.71%)	26 (3.96%)	29 (9.45%)	<.001
Antidiabetic medication	48 (4.98%)	22 (3.35%)	26 (8.47%)	<.001
Statins	111 (11.53%)	62 (9.45%)	49 (15.96%)	.003

BMI, body mass index; CAD, coronary artery disease; lAmygA, left amygdala activity; SNA, stress-related neural activity; vmPFC, ventromedial prefrontal cortex.

^a^Based on cut-points defined by a classification and regression tree analysis (CART) for time-to-event data with high lAmygA/vmPFC ≥ 0.727.

### Laboratory measurements and echocardiography findings

Within 6 months of ^18^F-FDG-PET, all patients underwent transthoracic echocardiography. Mean left ventricular ejection fraction (LVEF) was 58.0 ± 9.9%. Left ventricular hypertrophy was diagnosed in 103 patients (12.7%), while wall motion abnormalities, diastolic dysfunction, or valvular heart disease were detected in 6.9%, 28.5%, and 19.8% of patients, respectively (*Table 2*). Mean LVEF did not differ significantly between patients with and without cancer history (57.9 ± 8.2% vs. 58.1 ± 10.8%, *P* = .802). Circulating inflammatory markers comprising C-reactive protein, neutrophils, and lymphocytes, measured within 6 days and within 12 months of ^18^F-FDG-PET, respectively, are depicted in *Table 2*.

**Table 2:** Cardiac imaging findings and laboratory values within 6 (12) months of ^18^F-FDG-PET stratified by stress-related neural activity (lAmygA/vmPFC)

Patient’s characteristics	Total (*n* = 963)	SNA (lAmygA/vmPFC)		*P*-value
		Low^a^ (*n* = 656)	High^a^ (*n* = 307)	
Echocardiography/MRI findings				
LV hypertrophy	103 (12.68%)	67 (12.25%)	36 (13.58%)	.59
LVEF (%)—mean (SD)	58.00 (9.91)	58.32 (9.53)	57.34 (10.59)	.19
LV wall motion abnormalities	66 (6.85%)	36 (5.49%)	30 (9.77%)	.014
LV diastolic dysfunction	274 (28.45%)	173 (26.37%)	101 (32.90%)	.036
Valvular heart disease	191 (19.83%)	112 (17.07%)	79 (25.73%)	.002
Inflammation markers (within 6 days of ^18^F-FDG-PET)—median (IQR)				
CRP (mg/L)	6.90 (2.00–34.00)	4.15 (1.20–16.00)	9.90 (4.10–57.00)	<.001
WBC—neutrophiles (1000/μL)	3.92 (2.50–6.14)	3.47 (2.39–5.26)	4.85 (2.96–7.68)	<.001
WBC—lymphocytes (1000/μL)	1.22 (0.73–1.84)	1.20 (0.72–1.89)	1.28 (0.77–1.78)	.80
^18^F-FDG bone marrow uptake (SUVmax)	2.26 (0.68)	2.22 (0.61)	2.33 (0.81)	.040
^18^F-FDG splenic uptake (SUVmean)	1.81 (2.31)	1.71 (0.50)	2.01 (4.04)	.069
Inflammation markers (within 12 months of ^18^F-FDG-PET)—median (IQR**)**				
CRP (mg/L)	87 (20–186)	75 (15–170)	113 (32–214.00)	<.001
WBC—neutrophiles (1000/μL)	10.18 (6.60–16.09)	9.47 (6.22–15.87)	11.47 (7.54–16.35)	.003
WBC—lymphocytes (1000/μL)	2.00 (1.46–2.74)	1.98 (1.44–2.65)	2.09 (1.53–2.84)	.042
Laboratory values (within 12 months of ^18^F-FDG-PET)—median (IQR)				
Creatinine (μmol/L)	93 (78–132)	90 (76–118)	112 (84–189)	<.001
Non-fasting glucose (mmol/L)	5.50 (5.00–6.20)	5.30 (5.00–5.90)	5.90 (5.30–6.90)	<.001
NT-proBNP (ng/L)	544 (144–2879)	344 (100–1688)	1394 (269–6990)	<.001

^18^F-FDG-PET, 2-[^18^F]fluoro-2-deoxy-D-glucose positron emission tomography; CRP, C-reactive protein; lAmygA, left amygdala metabolic activity; LV, left ventricle; LVEF, left ventricular ejection fraction; MRI, magnetic resonance imaging; NT-proBNP, N-terminal pro-brain natriuretic peptide; SNA, stress-related neural activity; vmPFC, ventromedial prefrontal cortex; WBC, white blood cell count.

^a^Based on cut-points defined by a classification and regression tree analysis (CART) for time-to-event data with high lAmygA/vmPFC ≥ 0.727.

### Relation between stress-associated neural activity and adverse events

During follow-up, 118 patients (12.3%) experienced MACE, and 270 (28.0%) died. *Table 3* lists baseline variables in the overall study population stratified by study endpoints.

**Table 3:** Patient’s baseline characteristics and stress-related neural activity stratified by binary outcomes (major adverse cardiovascular events and all-cause mortality)

Factors	Total (*n* = 963)	MACE			All-cause mortality		
		MACE (*n* = 118)	No MACE (*n* = 845)	*P*-value	Death (*n* = 270)	Alive (*n* = 693)	*P*-value
Age (years)—mean (SD)	58.43 (16.07)	65.35 (12.57)	57.47 (16.28)	<.001	65.49 (12.78)	55.68 (16.39)	<.001
Sex				.11			.11
Male	571 (59.29%)	78 (66.10%)	493 (58.34%)		171 (63.33%)	400 (57.72%)	
Female	392 (40.71%)	40 (33.90%)	352 (41.66%)		99 (36.67%)	293 (42.28%)	
BMI (kg/m^2^)—mean (SD)	25.59 (5.10)	25.69 (4.89)	25.57 (5.13)	.84	25.16 (5.20)	25.74 (5.05)	.16
Heart rate (b.p.m.)—mean (SD)	77.34 (15.33)	76.66 (14.31)	77.44 (15.47)	.62	78.96 (15.79)	76.73 (15.12)	.052
Socioeconomic variables							
Living situation				.73			.043
Partnership/married	553 (57.42%)	66 (55.93%)	487 (57.63%)		169 (62.59%)	384 (55.41%)	
Living alone	410 (42.58%)	52 (44.07%)	358 (42.37%)		101 (37.41%)	309 (44.59%)	
Occupation skill level				.20			.002
Low	794 (87.54%)	104 (91.23%)	690 (87.01%)		238 (92.97%)	556 (85.41%)	
High	113 (12.46%)	10 (8.77%)	103 (12.99%)		18 (7.03%)	95 (14.59%)	
Comorbidities							
Comorbidities—non-cardiac	98 (10.18%)	14 (11.86%)	84 (9.94%)	.52	33 (12.22%)	65 (9.38%)	.19
Comorbidities—cardiac	340 (35.31%)	56 (47.46%)	284 (33.61%)	.003	111 (41.11%)	229 (33.04%)	.019
Known CAD	182 (18.90%)	26 (22.03%)	156 (18.46%)	.35	56 (20.74%)	126 (18.18%)	.36
Known structural or valvular heart disease, arrhythmia	215 (22.33%)	45 (38.14%)	170 (20.12%)	<.001	77 (28.52%)	138 (19.91%)	.004
History of cancer	355 (36.86%)	32 (27.12%)	323 (38.22%)	.019	79 (29.26%)	276 (39.83%)	.002
Chronic inflammatory disease	34 (3.53%)	4 (3.39%)	30 (3.55%)	.93	7 (2.59%)	27 (3.90%)	.32
Cardiovascular risk factors							
Obesity	134 (16.54%)	14 (14.89%)	120 (16.76%)	.65	32 (14.95%)	102 (17.11%)	.47
Diabetes	189 (19.65%)	38 (32.20%)	151 (17.89%)	<.001	64 (23.70%)	125 (18.06%)	.048
Dyslipidaemia	289 (30.04%)	55 (46.61%)	234 (27.73%)	<.001	77 (28.52%)	212 (30.64%)	.52
Hypertension	446 (46.36%)	71 (60.17%)	375 (44.43%)	.001	141 (52.22%)	305 (44.08%)	.023
Family history of CAD	127 (13.20%)	18 (15.25%)	109 (12.91%)	.48	27 (10.00%)	100 (14.45%)	.067
Smoking	492 (51.14%)	73 (61.86%)	419 (49.64%)	.013	138 (51.11%)	354 (51.16%)	.99
Medication							
Blood pressure/heart failure medication	214 (22.22%)	27 (22.88%)	187 (22.13%)	.85	82 (30.37%)	132 (19.05%)	<.001
Antiplatelet/anticoagulants	158 (16.41%)	20 (16.95%)	138 (16.33%)	.87	51 (18.89%)	107 (15.44%)	.19
Anti-inflammatory drugs	201 (20.87%)	21 (17.80%)	180 (21.30%)	.38	65 (24.07%)	136 (19.62%)	.13
Antiarrhythmics	17 (1.77%)	2 (1.69%)	15 (1.78%)	.95	8 (2.96%)	9 (1.30%)	.078
Antidepressants	55 (5.71%)	9 (7.63%)	46 (5.44%)	.34	21 (7.78%)	34 (4.91%)	.085
Antidiabetic medication	48 (4.98%)	7 (5.93%)	41 (4.85%)	.61	18 (6.67%)	30 (4.33%)	.13
Statins	111 (11.53%)	12 (10.17%)	99 (11.72%)	.62	38 (14.07%)	73 (10.53%)	.12
Echocardiography							
LV hypertrophy	103 (12.68%)	15 (15.96%)	88 (12.26%)	.31	40 (18.52%)	63 (10.57%)	.003
LVEF (%)—mean (SD)	57.99 (9.91)	55.32 (12.39)	58.40 (9.40)	.003	56.66 (11.93)	58.57 (8.82)	.013
LV wall motion abnormalities	66 (6.85%)	11 (9.32%)	55 (6.51%)	.26	30 (11.11%)	36 (5.19%)	.001
LV diastolic dysfunction	274 (28.45%)	49 (41.53%)	225 (26.63%)	<.001	89 (32.96%)	185 (26.70%)	.053
Valvular heart disease	191 (19.83%)	23 (19.49%)	168 (19.88%)	.92	61 (22.59%)	130 (18.76%)	.18
Inflammation markers (within 6 days of ^18^F-FDG-PET) median (IQR)							
CRP (mg/L)	6.90 (2.00–34.00)	12.00 (5.70–54.00)	6.60 (1.80–28.50)	.014	8.00 (3.00–51.00)	5.50 (1.50–27.00)	.011
WBC—neutrophils (1000/μL)	3.92 (2.50–6.14)	5.81 (3.20–7.59)	3.82 (2.44–5.77)	.013	4.34 (2.89–6.32)	3.75 (2.43–6.02)	.062
WBC—lymphocytes (1000/μL)	1.22 (0.73–1.84)	1.41 (0.82–1.83)	1.22 (0.72–1.84)	.59	1.19 (0.74–1.70)	1.26 (0.72–1.93)	.34
^18^F-FDG uptake bone marrow (SUVmax)—mean (SD)	2.26 (0.68)	2.07 (0.50)	2.28 (0.69)	.002	2.16 (0.80)	2.29 (0.62)	.014
Laboratory values (within 12 months of ^18^F-FDG-PET—median (IQR)							
Creatinine (μmol/L)	93 (78–132)	102 (82–157)	93 (77–130)	.035	113 (85–213)	89 (76–119)	<.001
Non-fasting glucose level (mmol/L)	5.50 (5.00–6.20)	5.80 (5.20–6.70)	5.40 (5.00–6.10)	.005	5.80 (5.20–6.60)	5.40 (5.00–6.00)	<.001
NT-proBNP (ng/L)	544 (144–2879)	1377 (375–4795)	472 (130–2582)	<.001	1684 (342–6754)	365 (102–1657)	<.001
SNA—continuous							
Bilateral SNA (meanAmygA/vmPFC)—mean (SD), continuous	0.69 (0.08)	0.70 (0.08)	0.69 (0.08)	.071	0.71 (0.08)	0.68 (0.08)	<.001
Right SNA (rAmygA/vmPFC)—mean (SD), continuous	0.68 (0.08)	0.69 (0.09)	0.68 (0.08)	.18	0.70 (0.08)	0.68 (0.08)	<.001
Left SNA (lAmygA/vmPFC)—mean (SD), continuous	0.70 (0.08)	0.72 (0.09)	0.70 (0.08)	.039	0.72 (0.08)	0.69 (0.08)	<.001
Bilateral SNA (meanAmygA/vmPFC)—high vs. low				.12			<.001
SNA (meanAmygA/vmPFC) < 0.719, low	672 (69.78%)	75 (63.56%)	597 (70.65%)		148 (54.81%)	524 (75.61%)	
SNA (meanAmygA/vmPFC) ≥ 0.719, high	291 (30.22%)	43 (36.44%)	248 (29.35%)		122 (45.19%)	169 (24.39%)	
Right SNA (rAmygA/vmPFC)—high vs. low				.27			<.001
SNA (rAmygA/vmPFC) < 0.722, low	725 (75.29%)	84 (71.19%)	641 (75.86%)		166 (61.48%)	559 (80.66%)	
SNA (rAmygA/vmPFC) ≥ 0.722, high	238 (24.71%)	34 (28.81%)	204 (24.14%)		104 (38.52%)	134 (19.34%)	
Left SNA (lAmygA/vmPFC)—high vs. low				.029			<.001
SNA (lAmygA/vmPFC) < 0.727, low	656 (68.12%)	70 (59.32%)	586 (69.35%)		138 (51.11%)	518 (74.75%)	
SNA (lAmygA/vmPFC) ≥ 0.727, high	307 (31.88%)	48 (40.68%)	259 (30.65%)		132 (48.89%)	175 (25.25%)	

^18^F-FDG-PET, 2-[^18^F]fluoro-2-deoxy-D-glucose positron emission tomography; AmygA, amygdala activity; BMI, body mass index; b.p.m., beats per minute; CAD, coronary artery disease; CRP, C-reactive protein; lAmygA, left amygdala metabolic activity; LV, left ventricle; LVEF, left ventricular ejection fraction; meanAmygA, mean amygdala metabolic activity; NT-proBNP, N-terminal pro-brain natriuretic peptide; rAmygA, right amygdala metabolic activity; SNA, stress-related neural activity; vmPFC, ventromedial prefrontal cortex; WBC, white blood cell count.

**Figure 2:**
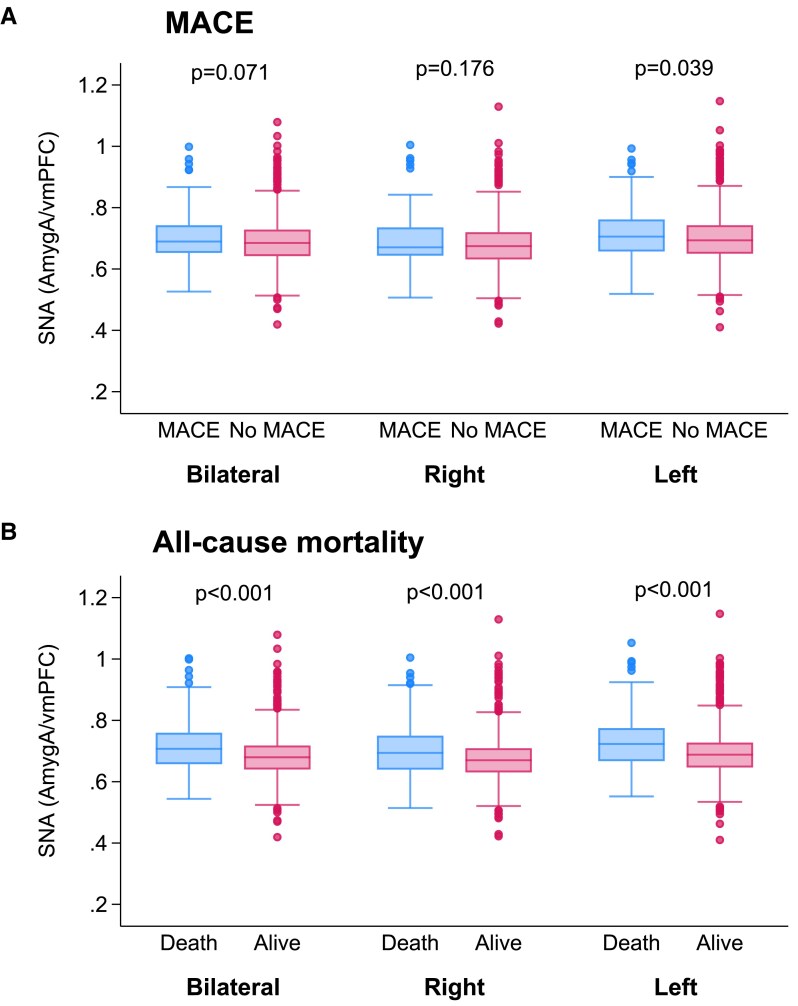
Analysis of SNA (AmygA/vmPFC) vs. study outcomes based on bilateral, right, and left amygdala activities, for (*A*) MACE, and (*B*) all-cause mortality. MACE, major adverse cardiovascular events; SNA, stress-related neural activity; vmPFC, ventromedial prefrontal cortex

Amongst the 270 patients who died, 238 (88%) died from non-cardiac causes, and 32 due to cardiac disease (11.8%). Stress-related neural activity (lAmygA/vmPFC) was significantly higher in patients who experienced MACE than in those who did not (*P* = .039) (*Figure 2A*, *Table 3*). In contrast, SNA (meanAmygA/vmPFC) and SNA (rAmygA/vmPFC) did not differ significantly between individuals with and without MACE (*P* = .071, and *P* = .176, respectively) (*Figure 2A*, *Table 3*).

**Figure 3:**
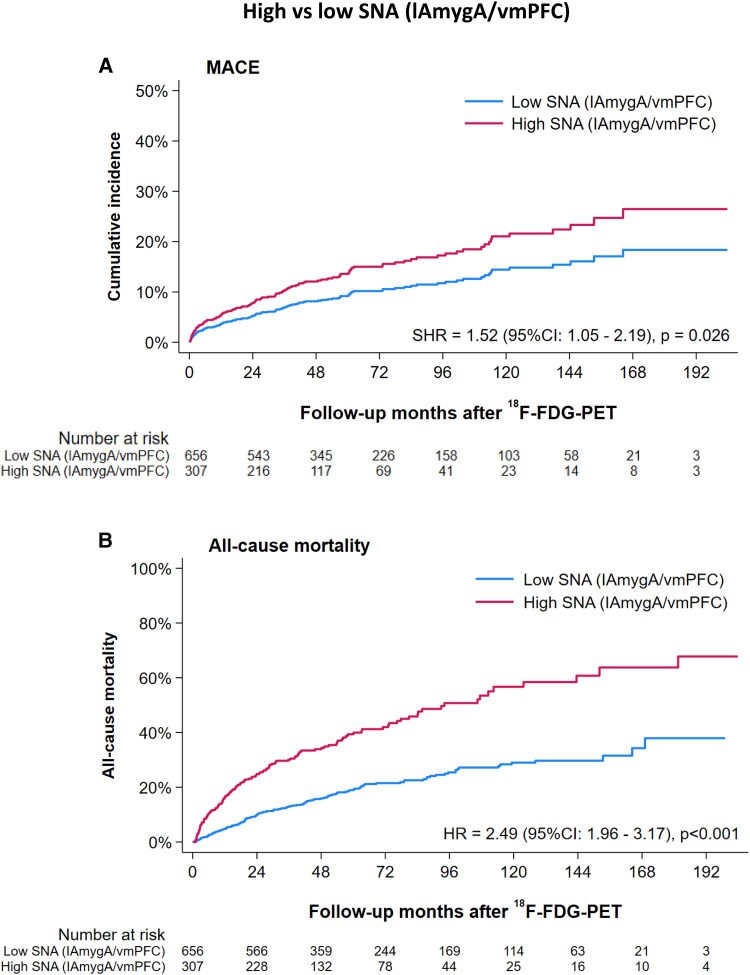
Unadjusted cumulative incidences of (*A*) MACE, and cumulative hazard of (*B*) all-cause mortality. ^18^F-FDG-PET, 2-[^18^F]fluoro-2-deoxy-D-glucose positron emission tomography; HR, hazard ratio; lAmygA, left amygdala activity; MACE, major adverse cardiovascular events; SHR, sub-distribution hazard ratio; SNA, stress-related neural activity; vmPFC, ventromedial prefrontal cortex

In patients who died from any cause during follow-up, SNA (meanAmygA/vmPFC), SNA (rAmygA/vmPFC), and SNA (lAmygA/vmPFC) were all significantly higher than in survivors (*P* < .001) (*Figure 2B*, *Table 3*). Based on CART analysis, individuals with SNA (meanAmygA/vmPFC) ≥ 0.719, SNA (rAmygA/vmPFC) ≥ 0.722, and SNA (lAmygA/vmPFC) ≥ 0.727 were defined as having high SNA and all others as having low SNA (*Table 3*). Given that dichotomized SNA (lAmygA/vmPFC) showed the strongest associations with study endpoints (*Table 3*), only SNA (lAmygA/vmPFC) was used for further analysis. Kaplan–Meier survival curves for MACE and all-cause mortality yielded significant group differences between individuals with high vs. low SNA (lAmygA/vmPFC) (*Figure 3*). We observed a 1.5-fold higher risk for MACE (sub-distribution hazard ratio [SHR] 1.52 [95% CI: 1.05–2.19], *P* = .026) (*Figure 3A*, *Table 4*) and a 2.5-fold higher risk for all-cause mortality (hazard ratio [HR] 2.49 [95% CI: 1.96–3.17], *P* < .001) (*Figure 3B*, *Table 4*) in the high SNA (lAmygA/vmPFC) group compared to the low SNA (lAmygA/vmPFC) group.

**Table 4:** Time-to-event analysis depicting the bivariate association between baseline variables, laboratory parameters, imaging findings, stress-related neural activity (lAmygA/vmPFC), and outcomes

Factors	MACE		All-cause mortality	
	Crude SHR (95% CI)	*P*-value	Crude HR (95% CI)	*P*-value
Age (years)	1.03 (1.02, 1.05)	<.001	1.04 (1.03, 1.05)	<.001
Sex (male vs. female)	1.38 (0.94, 2.02)	.097	1.24 (0.97, 1.58)	.093
BMI (kg/m^2^)	1.00 (0.97, 1.04)	.826	0.98 (0.95, 1.01)	.151
Heart rate (×10 b.p.m.)	0.97 (0.86, 1.09)	.556	1.09 (1.01, 1.17)	.030
Socioeconomic variables				
Living alone vs. living not alone	1.02 (0.71, 1.46)	.911	0.73 (0.57, 0.93)	.012
Low occupation skill level	1.41 (0.74, 2.69)	.300	1.95 (1.21, 3.15)	.006
Comorbidities				
Known comorbidities—non-cardiac	1.26 (0.72, 2.20)	.426	1.40 (0.97, 2.01)	.071
Comorbidities—cardiac	1.82 (1.27, 2.62)	.001	1.45 (1.14, 1.85)	.003
History of cancer	0.74 (0.49, 1.11)	.141	0.82 (0.63, 1.07)	.149
Chronic inflammatory disease	1.02 (0.37, 2.80)	.969	0.73 (0.35, 1.56)	.420
Cardiovascular risk factors				
Obesity	0.89 (0.50, 1.58)	.694	0.90 (0.62, 1.32)	.596
Diabetes	2.20 (1.49, 3.23)	<.001	1.46 (1.10, 1.93)	.009
Dyslipidaemia	2.22 (1.55, 3.19)	<.001	0.95 (0.73, 1.24)	.696
Hypertension	1.78 (1.24, 2.58)	.002	1.27 (1.00, 1.61)	.050
Family history of CAD	1.23 (0.75, 2.02)	.412	0.72 (0.48, 1.07)	.101
Smoking	1.74 (1.20, 2.51)	.004	1.10 (0.86, 1.39)	.456
Medication				
Blood pressure/heart failure	1.08 (0.70, 1.66)	.726	1.79 (1.38, 2.32)	<.001
Antiplatelet/anticoagulants	1.12 (0.69, 1.81)	.641	1.36 (1.003, 1.85)	.048
Anti-inflammatory drugs	0.89 (0.56, 1.43)	.629	1.41 (1.07, 1.87)	.016
Antiarrhythmics	1.00 (0.24, 4.12)	.999	2.00 (0.99, 4.05)	.054
Antidepressants	1.48 (0.74, 2.93)	.264	1.66 (1.06, 2.59)	.026
Antidiabetic medication	1.30 (0.60, 2.81)	.500	1.53 (0.95, 2.47)	.081
Statins	0.90 (0.49, 1.65)	.734	1.39 (0.98, 1.95)	.063
Echocardiography/MRI findings				
LV hypertrophy	1.34 (0.77, 2.33)	.299	1.75 (1.24, 2.47)	.001
LVEF (%)	0.97 (0.96, 0.99)	.001	0.98 (0.97, 0.99)	<.001
LV wall motion abnormalities	1.44 (0.77, 2.70)	.250	2.00 (1.37, 2.93)	<.001
LV diastolic dysfunction	1.84 (1.28, 2.65)	.001	1.27 (0.98, 1.63)	.068
Valvular heart disease	0.97 (0.61, 1.54)	.892	1.19 (0.90, 1.58)	.231
Inflammation markers (within 6 days of ^18^F-FDG-PET)				
CRP (mg/L)	1.001 (0.998, 1.005)	.467	1.002 (0.999, 1.005)	.313
WBC—neutrophiles (1000/μL)	1.049 (1.003, 1.097)	.038	1.044 (1.001, 1.089)	.044
WBC—lymphocytes (1000/μL)	0.989 (0.968, 1.011)	.319	1.009 (1.000, 1.019)	.052
Inflammation marker (within 12 months of ^18^F-FDG-PET)				
CRP (mg/L)	1.000 (0.999, 1.002)	.896	1.002 (1.001, 1.003)	<.001
WBC—neutrophils (1000/μL)	0.993 (0.978, 1.008)	.353	1.000 (0.991, 1.009)	.942
WBC—lymphocytes (1000/μL)	0.967 (0.905, 1.033)	.324	1.004 (0.995, 1.012)	.425
Lab values (within 12 months of ^18^F-FDG-PET)				
Creatinine (μmol/L)	1.001 (1.000, 1.002)	.124	1.002 (1.002, 1.003)	<.001
NT-proBNP (ng/L) × 10 000	1.124 (0.997, 1.267)	.055	1.208 (1.119, 1.305)	<.001
Non-fasting glucose (mmol/L)	1.013 (0.991, 1.036)	.251	1.026 (1.004, 1.048)	.018
^18^F-FDG-PET imaging parameters				
^18^F-FDG uptake bone marrow (SUVmax)	0.61 (0.43, 0.87)	.007	0.83 (0.67, 1.03)	.096
^18^F-FDG splenic uptake (SUVmean)	1.07 (1.06, 1.08)	<.001	0.89 (0.68, 1.17)	.410
High SNA (lAmygA/vmPFC)	1.52 (1.05, 2.19)	.026	2.49 (1.96, 3.17)	<.001

^18^F-FDG-PET, 2-[^18^F]fluoro-2-deoxy-D-glucose positron emission tomography; BMI, body mass index; b.p.m., beats per minute; CAD, coronary artery disease; CRP, C-reactive protein; HR, hazard ratio; LV, left ventricle; LVEF, left ventricular ejection fraction; NT-proBNP, N-terminal pro-brain natriuretic peptide; SHR, sub-distribution hazard ratio; WBC, white blood cell count.

### Correlation of baseline and clinical variables with stress-related neural activity (lAmygA/vmPFC)

Unadjusted linear regression analysis exploring the association between baseline/clinical parameters and SNA (lAmygA/vmPFC) revealed strong correlations of age (standardized *β* coefficient [95% CI] = .298 [0.244, 0.352], *P* < .001), hypertension (*β* = .181 [0.121, 0.240], *P* < .001), blood pressure/HF medication (*β* = .153 [0.092, 0.215], *P* < .001), inflammatory markers [C-reactive protein (*β* = .282 [0.186, 0.379], *P* < .001), neutrophils (*β* = .156 [0.044, 0.268], *P* = .006)], and creatinine (*β* = .155 [0.092, 0.219], *P* < .001) with SNA (lAmygA/vmPFC). A full list is provided in Supplementary data online, *Table S2*.

### Clinical variables according to stress-related neural activity (lAmygA/vmPFC)

To explore the association between SNA (lAmygA/vmPFC) and baseline characteristics, the population was stratified according to dichotomized (high vs. low) SNA (lAmygA/vmPFC). Individuals with high SNA (lAmygA/vmPFC) were significantly older (64.4 ± 13.2 years vs. 55.7 ± 16.5 years, *P* < .001) and had a higher prevalence of CVRFs and comorbidities than individuals with low SNA (lAmygA/vmPFC, *Table 1*). Moreover, individuals on medical therapies such as blood pressure/HF medication (30.3% vs. 18.5%, *P* < .001), antiplatelet/anticoagulant compounds (22.5% vs. 13.6%, *P* < .001), anti-inflammatory drugs (26.7% vs. 18.1%, *P* = .002), antidepressants (9.5% vs. 4.0%, *P* < .001), antidiabetic medication (8.5% vs. 3.4%, *P* < .001), or statins (16.0% vs. 9.5%, *P* = .003) were more frequently in the high SNA (lAmygA/vmPFC) than in the low SNA (lAmygA/vmPFC) group (*Table 1*).

Conversely, individuals with cancer history were more often in the low SNA (lAmygA/vmPFC) group than those without cancer history (40.2% vs. 29.6%, *P* = .001, *Table 1*), while there was no in-between-group difference in individuals with chronic inflammatory disease or according to sex, BMI, or socioeconomic status (*Table 1*).

### Echocardiography and laboratory parameters according to stress-related neural activity (lAmygA/vmPFC)

*Table 2* depicts group differences in echocardiography and laboratory parameters at the time of ^18^F-FDG-PET between individuals with high vs. low SNA (lAmygA/vmPFC). Compared to individuals with low SNA (lAmygA/vmPFC), individuals with high SNA (lAmygA/vmPFC) more often had left ventricular wall motion abnormalities (9.8% vs. 5.5%, *P* = .014), left ventricular diastolic dysfunction (32.9% vs. 26.4%, *P* = .036), or valvular heart disease (25.7% vs. 17.1%, *P* = .002) (*Table 2*). Further, individuals with high SNA (lAmygA/vmPFC) had higher levels of C-reactive protein within ±3 days from ^18^F-FDG/PET (9.9 [4.1–57.0] mg/L vs. 4.2 [1.2–16.0] mg/L, *P* < .001), neutrophils (4.9 [3.0–7.7]/µL vs. 3.5 [2.4–5.3]/µL, *P* < .001) as well as a trend towards higher bone marrow or splenic activity, indicated by ^18^F-FDG uptake, (bone marrow: 2.33 ± 0.81 SUVmax vs. 2.22 ± 0.61 SUVmax, *P* = 0.04; spleen: 2.01 ± 4.04 SUVmean vs. 1.71 ± 0.50 SUVmean, *P* = .069) than individuals with low SNA (lAmygA/vmPFC). Creatinine levels (112 [84–189] µmol/L vs. 90 [76–118] µmol/L, *P* < .001), non-fasting glucose (5.9 [5.3–6.9] mmol/L vs. 5.3 [5.0–5.9] mol/L, *P* < .001), and NT-proBNP (1394 [269–6990] ng/L vs. 344 [100–1688] ng/L, *P* < .001) were all significantly higher in the high SNA (lAmygA/vmPFC) group compared to the low SNA (lAmygA/vmPFC) group (*Table 2*).

### Unadjusted association of baseline and imaging variables with study endpoints

The association between baseline variables, SNA (lAmygA/vmPFC), and study outcomes was assessed using Fine and Gray’s proportional sub-distribution hazards models for MACE and Cox regression models for all-cause mortality. Unadjusted associations between all baseline and imaging variables with study endpoints are depicted in *Table 4*.

### Prognostic value of stress-related neural activity (lAmygA/vmPFC) for the prediction of major adverse cardiovascular events and all-cause mortality

**Figure 4:**
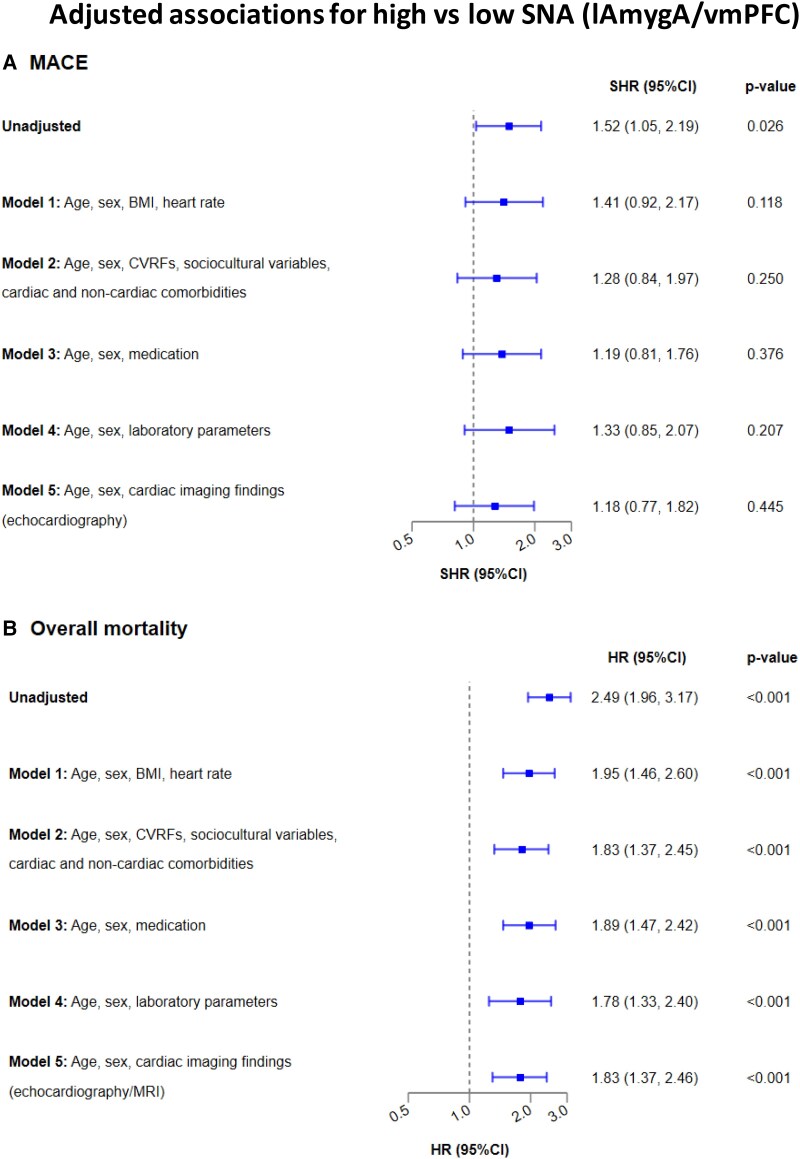
Multivariate analysis adjusted for baseline clinical characteristics, laboratory measures, and cardiac imaging findings, for (*A*) MACE, and (*B*) all-cause mortality. BMI, body mass index; CVRF, cardiovascular risk factor; HR, hazard ratio; MACE, major adverse cardiovascular events; SHR, sub-distribution hazard ratio

The association between SNA (lAmgyA/vmPFC) and the primary study endpoint MACE was lost when information about basic demographic variables (age, sex, BMI, and heart rate, Model 1: adjusted SHR 1.41 [95% CI: 0.92–2.17], *P* = .118), age, sex, CVRFs, sociocultural variables, and comorbidities (Model 2: adjusted SHR 1.28 [95% CI: 0.84–1.97], *P* = .250), age, sex, and medication (Model 3: adjusted SHR 1.19 [95% CI: 0.81–1.76], *P* = .376), age, sex, and laboratory parameters (Model 4: adjusted SHR 1.33 [95% CI: 0.85–2.07], *P* = .207), or age, sex, and echocardiography findings (Model 5: adjusted SHR 1.18 [95% CI: 0.77–1.82], *P* = .445) were added to the model (*Figure 4A*).

Conversely, the relationship between SNA (lAmgyA/vmPFC) and the secondary outcome of all-cause mortality remained robust after multivariable adjustments in all five models (*Figure 4B*). In fact, SNA (lAmgyA/vmPFC) remained a strong and independent predictor of all-cause mortality after adjustment for demographic variables (Model 1: adjusted HR 1.95 [95% CI: 1.46–2.60], *P* < .001), or when adding to age and sex CVRFs, sociocultural variables, and comorbidities (Model 2: adjusted HR 1.83 [95% CI: 1.37–2.45], *P* < .001), medication (Model 3: adjusted HR 1.89 [95% CI: 1.47–2.42], *P* < .001), laboratory parameters (Model 4: adjusted HR 1.78 [95% CI: 1.33–2.40], *P* < .001), or echocardiography findings (Model 5: adjusted HR 1.83 [95% CI: 1.37–2.46], *P* < .001). The full models are depicted in *Table 5*.

**Table 5:** Associations between stress-related neural activity (lAmygA/vmPFC) and outcomes: multivariable analysis with adjustment for various confounding factors

	MACE		All-cause mortality	
	Adjusted SHR (95% CI)	*P*-value	Adjusted HR (95% CI)	*P*-value
Model 1: age, sex, BMI, and heart rate				
SNA (lAmygA/vmPFC): per unit change	3.00 (0.26, 34.79)	.381	7.48 (1.51, 37.19)	.014
SNA (lAmygA/vmPFC): high vs. low^a^	1.41 (0.92, 2.17)	.118	1.95 (1.46, 2.60)	<.001
Model 2: age, sex, CVRFs, cardiac and non-cardiac comorbidities, and sociocultural variables				
SNA (lAmygA/vmPFC): per unit change	2.33 (0.18, 30.20)	.517	4.57 (0.85, 24.54)	.076
SNA (lAmygA/vmPFC): high vs. low^a^	1.28 (0.84, 1.97)	.250	1.83 (1.37, 2.45)	<.001
Model 3: age, sex, and medication				
SNA (lAmygA/vmPFC): per unit change	2.44 (0.33, 18.31)	.385	6.14 (1.67, 22.50)	.006
SNA (lAmygA/vmPFC): high vs. low^a^	1.19 (0.81, 1.76)	.376	1.89 (1.47, 2.42)	<.001
Model 4: age, sex, and laboratory parameters				
SNA (lAmygA/vmPFC): per unit change	1.52 (0.15, 15.05)	.722	3.74 (0.88, 15.90)	.074
SNA (lAmygA/vmPFC): high vs. low^a^	1.33 (0.85, 2.07)	.207	1.78 (1.33, 2.40)	<.001
Model 5: age, sex, and cardiac imaging findings (echocardiography)				
SNA (lAmygA/vmPFC): per unit change	1.95 (0.15, 25.83)	.612	7.97 (1.55, 40.90)	.013
SNA (lAmygA/vmPFC): high vs. low^a^	1.18 (0.77, 1.82)	.445	1.83 (1.37, 2.46)	<.001

BMI, body mass index; CVRF, cardiovascular risk factor; HR, hazard ratio; lAmygA, left amygdala metabolic activity; MACE, major adverse cardiovascular events; SHR, sub-distribution hazard ratio; SNA, stress-related neural activity; vmPFC, ventromedial prefrontal cortex.

^a^Cut-point defined by classification and regression tree analysis (CART) with high lAmygA/vmPFC ≥ 0.727.

Notably, similar associations between SNA and MACE or SNA and all-cause mortality were observed in multivariable models when SNA was normalized to the temporal lobe (lAmygA/temp) instead of the vmPFC. These data are presented in Supplementary data online, *Table S3*. The association between SNA (lAmgyA/vmPFC) and all-cause mortality was stronger than the one between SNA (lAmygA/temp) and all-cause mortality, as evidenced by *C*-statistics.

## Discussion

Previous studies have provided important insights into the mechanisms linking psychological stress to MACE.^7,8^ Notably, the SNA axis emerges as a target for pharmacologic or non-pharmacologic (e.g. stress reduction techniques) approaches to interrupt transmission along it. Consequently, SNA imaging using ^18^F-FDG-PET could guide CV risk stratification and/or monitoring of therapeutic interventions.^14,17^ However, the value of SNA imaging in the presence of routinely obtained parameters in CV care is currently unknown. To address this issue, we evaluated the incremental long-term prognostic value of SNA imaging over detailed clinical, biological, and echocardiography findings in a real-world European scenario. Our longitudinal analysis, conducted in a well-characterized, large population with extended follow-up, is the first to show that the association between SNA and MACE is lost when details of the CV history, laboratory parameters, cardiac imaging findings, or sociocultural characteristics are known. Conversely, the relationship between SNA and all-cause mortality remains robust after multivariable adjustments. Our data emphasize that age, comorbidities, medical therapies, and inflammation modify the effect of SNA on CV endpoints (*Structured Graphical Abstract*). These findings, thus, provide insights into the clinical value of SNA imaging in a real-world setting consisting of typical frail and older CV patients.

While our results confirm previous evidence reporting an influence of age,^23,34^ socioeconomic and lifestyle variables^6,8–10,14^, CVD risk factors and disease states,^14,17–20^ inflammation,^7,17,35^ or pharmacological treatments^4,21,36^ on SNA, our findings diverge from prior investigations showing an independent association between SNA and MACE.^5,7,8^ Several explanations for this apparent contradiction can be considered. First, to the best of our knowledge, this is the first study investigating the prognostic role of SNA in a European population, while other studies included U.S.^4,6,7,9–11,17,18^ and South Korean^13,14,20^ populations. Geographical differences may be relevant as socio-environmental factors such as noise exposure and socioeconomic disparities increase CV mortality through enhanced SNA,^8,10^ and could partly explain the heavier CV burden of SNA in these countries compared to Switzerland.^37^ Second, our population is unique in terms of inclusion criteria and well-characterized health status. Participants in previous studies were healthier,^5–9,17^ attributes rarely seen in clinical cardiology, thereby limiting these findings’ generalizability. Conversely, we have investigated patients with conditions affecting glucose metabolism and SNA, including medical therapies, systemic inflammation, active comorbidities, and cardiac conditions, and have controlled for these variables in our models. Notably, our study cohort comprised patients with reduced LVEF and rhythm disorders, who were recently shown to have reduced, and not increased, AmygA.^12^ Additionally, the Swiss Federal Department for Health Affairs covers a variety of ^18^F-FDG-PET indications exceeding oncological contexts.^38^ Thus, our population could reflect a wider variety of clinical scenarios and better depict the prognostic value of SNA imaging in a real-world setting. Third, methodological differences regarding MACE definition might have partly accounted for the weaker association between SNA and MACE seen in our analysis, with previous studies using the less restrictive Framingham Heart Study criteria.^6,39^ Fourth, differential definitions of SNA might account for the observed inconsistencies between studies. While we used the lAmygA/vmPFC ratio to reflect SNA, thereby capturing a potential cardioprotective effect of the brain missed by other approaches,^6,11^ most previous reports defined SNA as the ratio between AmygA and metabolic activities in other brain regions such as the temporal lobe or the cerebellum.^5,7–10,18^ Nevertheless, analysis of our data by using SNA (lAmygA/temp) instead of SNA (lAmygA/vmPFC) yielded similar results.

Unlike in previous studies where ICD codes were used to identify MACE,^6^ we have thoroughly assessed outcomes by systematically performing phone calls, consulting clinical records, and interrogating death registries. This rigorous approach allowed us to obtain a more complete assessment and reduced the risk of event misclassification. Similarly, socioeconomic factors were evaluated at an individual level and not derived from zip code or neighbourhood status.^8^ Our results suggest that the prognostic value of SNA imaging for MACE may hold true before the development of CVDs and less so after CVDs have occurred. If confirmed, this hypothesis would highlight the importance of initiating early preventive measures to reduce SNA before CVD onset, such as meditation or physical exercise.^14,40,41^

Another finding of our study is that SNA remains predictive of all-cause mortality even after correcting for baseline clinical, biological, and cardiac structural abnormalities. To the best of our knowledge, the association of SNA with all-cause mortality has not yet been explored. Indeed, given the wealth of data linking CV health to stress, previous studies have focused exclusively on the impact of SNA on MACE.^5–8^ Yet, stress impairs health beyond CVDs, with a greater risk of death, including from unnatural causes, in patients with stress-related disorders.^42^ The widening survival curves over time for high vs. low SNA and the robustness of the association between high SNA and all-cause mortality following multivariable adjustment highlights the strength of this link.

Currently, there is no consensus on how to quantify SNA using ^18^F-FDG-PET. However, approaches to correct AmygA to a remote cerebral activity, such as the cerebellum, the temporal lobes, or the vmPFC, have increasingly been applied.^4,7,11^ Normalizing to remote tissue activity helps to overcome the inter-variability issues introduced in SUV measurement by differences in injected radiotracer activities, PET/CT camera characteristics, and inter-individual metabolism.^43^ Accordingly, the European Association of Nuclear Medicine recommends using semi-quantitative measures, i.e. ‘SUV relative to a normal brain region’.^43^ The rationale behind choosing the cerebellum and the temporal lobes is that these regions are not involved in the neural stress loop and reliably depict baseline brain activity. Conversely, the vmPFC’s role in attenuating stress responses is increasingly acknowledged.^44–46^ As such, the ratio of AmygA and vmPFC metabolic activity is thought to reflect an interaction between functionally connected neural regions that promote vs. regulate the stress response. Notably, in our study, both SNA corrected for vmPFC metabolic activity as well as SNA corrected for temporal lobe metabolic activity produced comparable results. While these findings underline the reproducibility of measurements across different brain regions, it is notable that SNA (lAmygA/vmPFC) showed a stronger association with all-cause mortality than SNA (lAmygA/temp). This observation is consistent with previous studies, where using vmPFC activity to adjust for AmygA yielded a stronger association between SNA and clinical outcomes than adjusting for other background cerebral activity.^11^ Thus, we present novel evidence for the detrimental role of an imbalance in the AmygA/vmPFC interplay resulting in excess mortality. It should also be noted that the prefrontal cortex itself might directly affect CV health, independently from the amygdala. A recent study focusing on the rostromedial prefrontal cortex (rmPFC), a region adjacent to the vmPFC, in CAD patients, showed that a higher increase in rmPFC activity after acute mental stress was associated with a higher risk of MACE.^28^ The independent involvement of the prefrontal cortex in mental stress-associated CV outcomes warrants further study.

Furthermore, there is currently a lack of clarity regarding the significance of brain laterality when studying SNA.^47^ Our study is the first to present a comparative analysis between bilateral and unilateral AmygA and their association with MACE. We found a stronger association between lAmygA and MACE or overall mortality than for meanAmygA and rAmygA. This apparent laterality in SNA and its adverse impact is novel and robustly supported by previous reports suggesting a more prevalent role of left amygdala in emotion processing.^48–51^

An unexpected finding of our analysis was that, although HTA in the bone marrow and SNA were positively correlated, bone marrow activity and our study endpoints were inversely correlated. Conversely, a positive association was observed between splenic activity and clinical outcomes. Accordingly, increased splenic ^18^F-FDG uptake, but not bone marrow ^18^F-FDG uptake, was associated with an increased MACE risk in patients with a recent myocardial infarction.^52^ This finding can be attributed to the spleen’s larger leucocyte reservoir and its activation state, which may more closely reflect the pro-inflammatory state of circulating leucocytes.^52,53^ Additionally, ^18^F-FDG uptake in the vertebrae might not solely reflect bone marrow activity but also the activity of osteoblasts, stromal cells, and mesenchymal/haematopoietic stem cells.^54^ Moreover, although previous mediation analyses have convincingly shown that enhanced bone marrow activity mediated the effect of SNA on atherosclerosis and MACE, a direct effect of bone marrow activity on arterial inflammation and/or CV outcomes was missing.^5,7,13^ Such a direct link between enhanced bone marrow activity and arterial inflammation was recently shown in healthy individuals, suggesting that bone marrow activity is an early phenomenon contributing to atherosclerosis initiation and progression.^35^ Accordingly, in type I diabetic patients with more advanced atherosclerotic disease, a negative correlation between bone marrow activity and osteoprotegerin, a pro-inflammatory cytokine, was recently observed.^55^ Similarly, bone marrow activity did not correlate with arterial or systemic inflammation in stroke survivors,^13^ during an acute coronary syndrome,^56^ following anti-inflammatory treatment,^20^ and showed alterations over time.^4,57^ Observational and preclinical studies have also reported that aging correlates inversely with osteogenesis and bone haematopoiesis within bone.^58^ Altogether, these inconsistencies indicate that, albeit critical, HTA is merely one link in the complex loop bridging SNA and CV outcomes, subjected to high variability depending on age and disease state.

Intriguingly, we have observed in our cohort that patients with cancer history were more often in the low SNA (lAmygA/vmPFC) group than individuals without cancer. This seemingly paradoxical finding highlights the complexity of factors interfering with SNA. A recent study in breast cancer survivors showed a negative correlation between received social support, SNA, and inflammation, with left amygdala reactivity and C-reactive protein levels lowering as social support increases.^50^ Similarly, a reduced SNA might improve cancer survivors’ outcomes by lowering systemic inflammation,^59^ a central element of the brain–heart axis.^2^ Moreover, cancer is a traumatic experience resembling post-traumatic stress disorders.^60^ Consequently, the amygdala volume in cancer survivors with intrusive recollection is reduced, which might, in turn, affect SNA intensity.^61^ Furthermore, anticancer treatments such as radiation could have altered SNA in this subgroup.^62^ Lastly, this unexpected result could indicate a selection bias in our cohort, corresponding to a subgroup with lower SNA. Indeed, in a recent study on patients referred for cancer staging, SNA independently predicted mortality and cancer progression, with significantly lower progression-free survival in the upper SNA tertile group than in control patients.^63^

Our study has several limitations. First, baseline clinical information was obtained from the hospital’s database of patients who underwent clinically indicated ^18^F-FDG-PET (mainly evaluation of newly discovered lesions or cancer monitoring/follow-up), thus possibly limiting our results’ generalizability. Nonetheless, the association between SNA and outcomes remained significant in the non-oncological subgroup, suggesting that this has not affected our main results. Second, while we are the first to study the association between SNA and adverse outcomes in a European, mainly Caucasian, population, results obtained from a Swiss study sample might not be applied to other geographical regions given the impact of the socioeconomic environment on SNA.^8,9^ Third, lifestyle factors such as alcohol consumption and physical exercise that modify SNA could not be obtained in our study.^6,14^ Fourth, although we report the prevalence of depression in our sample, perceived stress was not assessed. Thus, the relation between emotional status, SNA, and adverse outcomes could not be analysed. Fifth, the number of all-cause mortality events in our population is significantly higher than the one of MACE, resulting in potential differences in the power analysis. Nevertheless, the MACE event rate in our study is higher than in comparable studies,^6–10^ making it unlikely that a lack of power accounted for the missing link between SNA and MACE. Finally, the risk of type 1 error occurring cannot be excluded, given the large number of conducted statistical tests. These limitations are substantially counterbalanced by several important innovations, including the unique data obtained from a large, real-world European study sample, the novel finding of laterality in SNA, as well as the observed robust association between SNA and all-cause mortality.

By illuminating that older individuals with CVDs profit less from SNA imaging, our study suggests that the prognostic value of SNA for MACE prediction largely depends on the population. Our results, together with previous reports, indicate that SNA imaging might be useful in a primary prevention setting in younger and healthier individuals with CVRFs but less so in secondary prevention. In this context, it will be important to determine the impact of aging itself and age-associated conditions on SNA. Conversely, SNA imaging may prove useful in patients with established CVD to predict all-cause mortality prior to high-risk procedures such as transcatheter aortic valve replacement. Hence, future studies need to determine which patient subgroups could benefit from routine SNA imaging as a diagnostic and/or monitoring tool. The question also arises at what stage SNA is still modifiable (timing hypothesis) and when active interventions, such as stress reduction techniques or pharmacological targeting of SNA, may be an effective prevention or treatment for CVD. Finally, the role of the prefrontal cortex in exerting potential cardioprotective effects needs to be further defined in future studies.

## Supplementary Material

ehae162_Supplementary_Data

## Declarations

### Data Availability

Based on the Business Administration System for Ethics Committees (BASEC) ethics approval, the non-anonymized raw data cannot be shared publicly. However, anonymized data that underlie the results reported in this article will become available to interested parties for non-commercial reasons after the publication upon reasonable requests made to the corresponding author. Data requestors will need to sign a data access agreement.

### Pre-registered Clinical Trial Number

None supplied.
