# Supplementary material for: Imaging of the brain–heart axis: prognostic value in a European setting
Source: Eur Heart J. 2024 Apr 10;45(18):1613–30. doi: 10.1093/eurheartj/ehae162 (PMC11089334; doi:10.1093/eurheartj/ehae162)
Supplement: ehae162_Supplementary_Data [file ehae162_supplementary_data.zip › 5_Supplementary Material_Revision.docx]

**Supplementary Methods**

**Measurement of hematopoietic tissue activity**

Metabolic activities in hematopoietic systems, such as the spleen and bone marrow, are well-known surrogate markers reflecting systemic inflammation. Hematopoietic tissue activity (HTA) was measured according to previously validated methods [^1^](#_ENREF_1). Briefly, ^18^F-FDG uptake was measured in the thoracic and lumbar vertebra (T1-L5 or T1-L4 in case of sacralization of L5) by placing ROIs encompassing the contour of each vertebra with the exclusion of the cortical bone. The SUVmax was determined for these regions, and total HTA was reported as the average SUVmax of the individual’s vertebrae. HTA was also calculated as the average of L3 and L4 vertebral SUVmax, as previously described [^2^](#_ENREF_2). Metabolic activity in the spleen was calculated for each patient by drawing ROIs in the spleen’s three orthogonal planes, i.e., the axial, sagittal, and coronal planes. The SUVmean was measured on each axis, and the mean of the three SUVmean values was calculated, as described previously [^3^](#_ENREF_3).

**Echocardiography**

Transthoracic echocardiography examinations were performed within 6 months of ^18^F-FDG-PET imaging at the University Hospital Zurich. Echocardiographic examinations were performed at the Department of Cardiology at the University Hospital Zurich using standardized protocols [^4^](#_ENREF_4)^,^[^5^](#_ENREF_5). The following left ventricle 2-dimensional derived parameters were calculated according to the biplane Simpson approach: end-diastolic volume (LVEDV), end-systolic volume (LVESV), and ejection fraction (LVEF). In addition, the presence of regional wall motion abnormalities was assessed visually. The cardiac valves were examined to study their anatomical integrity and identify pathological manifestations. LV mass was calculated by summation of the myocardial area multiplied by slice thickness plus image gap in the end-diastolic phase multiplied by the myocardial specific gravity (1.05g/mL). LVEF was calculated by dividing LVSV with LVEDV and multiplying by 100.

**Laboratory parameters**

Laboratory parameters, including inflammatory biomarkers such as C-reactive protein (CRP) levels, neutrophils, and lymphocytes, as well as renal function tests, blood glucose, and serum levels of NT-proBNP were retrieved from hospital electronic medical records for each patient. Only laboratory parameters obtained within 12 months (6 days for inflammatory markers) from the date of the ^18^F-FDG-PET imaging were included in the study.

**Statistical approach**

Descriptive statistics are reported as mean and standard deviation (SD) or median and interquartile range (25^th^ – 75^th^ percentile) for continuous variables, depending on the data distribution. To compare baseline characteristics between patients with high and low SNA (lAmgyA/vmPFC), or between those who experienced the study endpoints (major adverse cardiovascular events [MACE] and all-cause mortality), we performed a Chi-square test for categorical variables and an independent t-test of Mann-Whitney U test for continuous variables, depending on the distribution. Correlation between baseline or clinical parameters and SNA (lAmygA/vmPFC) was explored using unadjusted linear regression models. To identify cut-points in SNA (lAmgyA/vmPFC and lAmygA/temp), we performed a classification and regression tree analysis (CART) for time-to-event data based on the all-cause mortality endpoint using the *cart* command in Stata MP/18. [^6^](#_ENREF_6) Bivariate and multivariable analyses were performed to investigate the association between SNA (lAmgyA/vmPFC) (as continuous and as dichotomized variables [high vs. low based on CART analysis]) and time-to-event outcomes, where SNA (lAmgyA/vmPFC) had a linear effect on time-to-MACE and time-to-death. Time-to-event was defined as the time lag between the date of ^18^F-FDG-PET imaging and the date of death, date of MACE, or censored at the last follow-up date. Fine and Gray’s proportional sub-distribution hazards models were performed for MACE, assuming that non-cardiovascular death was a competing risk. Cumulative incidence for MACE and Kaplan-Meier failure curves for all-cause mortality are presented, Cox regression models were used to assess the association between SNA (lAmgyA/vmPFC) and all-cause mortality. The proportional hazard assumption for each variable was verified using the Schoenfeld residuals. To explore the impact of competing variables on the association between SNA (lAmgyA/vmPFC) and our study endpoint, we built five different models, where the covariables were defined *a priori*: we first evaluated the impact of basic demographic variables on the association between SNA (lAmgyA/vmPFC) and MACE/all-cause mortality (*Model 1*). Next, we assessed the effect of CVRFs, cardiac and non-cardiac comorbidities, as well as sociocultural variables on the association between SNA (lAmgyA/vmPFC) and MACE/all-cause mortality (*Model 2*), followed by medication *(Model 3*), laboratory parameters (*Model 4*), and cardiac imaging findings (*Model 5*). Accounting for the number of events, a maximum of 12 factors were considered in the models. Each of the five models included age and sex as fixed confounding factors. The missing rate was 2% - 20% for basic demographic, sociocultural, laboratory, and cardiac imaging findings variables. Therefore, missing data were dropped without applying the imputation method, and the number of cases included in the model was reported. Statistical testing was done within an exploratory framework at a two-sided significance level of α=0.05 without adjustment for multiple testing. All the statistical tests were performed using Stata MP/18 (StataCorp, 2023, College Station, TX, USA).

**References Supplementary Methods**

1. Fiechter M, Roggo A, Burger IA, Bengs S, Treyer V, Becker A*, et al.* Association between resting amygdalar activity and abnormal cardiac function in women and men: a retrospective cohort study. *Eur Heart J Cardiovasc Imaging* 2019;**20**:625-632. doi: 10.1093/ehjci/jez047

2. Devesa A, Lobo-González M, Martínez-Milla J, Oliva B, García-Lunar I, Mastrangelo A*, et al.* Bone marrow activation in response to metabolic syndrome and early atherosclerosis. *Eur Heart J* 2022;**43**:1809-1828. doi: 10.1093/eurheartj/ehac102

3. Emami H, Singh P, MacNabb M, Vucic E, Lavender Z, Rudd JH*, et al.* Splenic metabolic activity predicts risk of future cardiovascular events: demonstration of a cardiosplenic axis in humans. *JACC Cardiovasc Imaging* 2015;**8**:121-130. doi: 10.1016/j.jcmg.2014.10.009

4. Evangelista A, Flachskampf F, Lancellotti P, Badano L, Aguilar R, Monaghan M*, et al.* European Association of Echocardiography recommendations for standardization of performance, digital storage and reporting of echocardiographic studies. *Eur J Echocardiogr* 2008;**9**:438-448. doi: 10.1093/ejechocard/jen174

5. Lang RM, Badano LP, Mor-Avi V, Afilalo J, Armstrong A, Ernande L*, et al.* Recommendations for cardiac chamber quantification by echocardiography in adults: an update from the American Society of Echocardiography and the European Association of Cardiovascular Imaging. *Eur Heart J Cardiovasc Imaging* 2015;**16**:233-270. doi: 10.1093/ehjci/jev014

6. Putten W. Classification And Regression Tree analysis (CART) with Stata. *Stata Users Group, Dutch-German Stata Users' Group Meetings 2002* 2002. doi:
