# Supplementary material for: Imaging of the brain–heart axis: prognostic value in a European setting
Source: Eur Heart J. 2024 Apr 10;45(18):1613–30. doi: 10.1093/eurheartj/ehae162 (PMC11089334; doi:10.1093/eurheartj/ehae162)
Supplement: ehae162_Supplementary_Data [file ehae162_supplementary_data.zip › 5_Supplementary Table1.docx]

**Supplementary Table 1.** List of collected variables and main source of variable collection. ^18^F-FDG-PET/CT, 2-[18F]fluoro-2-deoxy-D-glucose positron emission tomography/computed tomography; ACC, American College of Cardiology; AHA, American Heart Association; AmygA, amygdala metabolic activity; BMI, body-mass-index; CAD, coronary artery disease; CRP, C-reactive protein; eGFR, estimated glomerular filtration rate; LV, left ventricle; LVEF, left ventricular ejection fraction; MACE, major adverse cardiovascular events; NSAID, non-steroidal anti-inflammatory drug; NT-proBNP, N-terminal pro-brain natriuretic peptide; SNA, stress-related neural activity; SUV, standard uptake value; vmPFC, ventromedial prefrontal cortex; WBC, white blood cell counts.

| **Collected data** | **Definition** | **Main source** |
| --- | --- | --- |
| **Demographic information** | | |
| Civil status | - | Phone calls |
| Professional skill level | As per the International Standard Classification of Occupations | Phone calls |
| **Comorbidities** | | |
| Non-cardiac | Documented history of cancer or chronic inflammatory diseases | Medical charts |
| Cardiac | Documented history of coronary artery disease, valvular and structural heart disease, heart failure, or rhythm disturbances | Medical charts |
| **Cardiovascular risk factors** | | |
| Obesity | BMI ≥30 kg/m^2^ (based on weight and height measured on 18F-FDG-PET day) | ^18^F-FDG-PET report |
| Diabetes mellitus | Documented history of diabetes with or without use of diabetes medications | Medical charts |
| Dyslipidemia | Documented history of dyslipidaemia with or without use of medications | Medical charts |
| Hypertension | Systolic blood pressure ≥140 mmHg or diastolic blood pressure ≥90 mmHg, or self-reported use of anti-hypertensive medication | Medical charts |
| Family history of CAD | Documented history of CAD in a first-degree male relative (i.e., father or brother) by age 55, or a first-degree female relative (i.e., mother or sister) by age 65 | Medical charts |
| Smoking | An adult who has smoked 100 cigarettes in his or her lifetime and who currently smokes cigarettes | Medical charts |
| **Medication** | | |
| Cardiovascular  medication | Blood pressure medication, heart failure medication, antiplatelet/anticoagulants, antiarrhythmics, statins, antidiabetic medication | Medical charts |
| Anti-inflammatory drugs | NSAID, corticosteroids, or immunosuppressive medication | Medical charts |
| Antidepressants | - | Medical charts |
| **Cardiac imaging findings** | | |
| LV hypertrophy | LV mass index >95g/m^2^ in women and >115 g/m^2^ in men | Echocardiography report |
| LVEF | LVEF = (LV end-systolic volume - LV end-diastolic volume) / (LV end-diastolic volume) | Echocardiography report |
| LV wall motion abnormalities | LV segmental hypokinesia, akinesia, or dyskinesia | Echocardiography report |
| LV diastolic dysfunction | Abnormalities of LV diastolic distensibility, filling, or relaxation, independent of the ejection fraction | Echocardiography report |
| Valvular heart disease | ≥ stage B valvulopathy according to the 2020 ACC/AHA Guideline for the management of patients With valvular heart disease | Echocardiography report |
| **Lab values (within 6 days and within 12 months of ^18^F-FDG-PET)** | | |
| Inflammation markers | WBC (neutrophils, lymphocytes), CRP | Lab charts |
| Renal function | Creatinine (umol/L)  Renal failure defined as eGFR ≤60 mL/min/1.73m^2^ | Lab charts |
| Cardiac biomarker | NT-proBNP | Lab charts |
| Metabolic marker | Non-fasting glucose | Lab charts |
| **^18^F-FDG-PET/CT imaging parameters** | | |
| SNA | AmygA/vmPFC | ^18^F-FDG-PET |
| Hematopoietic tissue activity | Bone marrow activity, by averaging SUV in bone marrow | ^18^F-FDG-PET |
| **Outcome** | | |
| MACE | Non-fatal stroke, non-fatal myocardial infarction, coronary revascularization, and cardiovascular death | Phone calls and death registries |
| All-cause mortality | - | Phone calls and death registries |
| **Other** | | |
| Heart rate | - | Echocardiography report |
