# Supplementary material for: Imaging of the brain–heart axis: prognostic value in a European setting
Source: Eur Heart J. 2024 Apr 10;45(18):1613–30. doi: 10.1093/eurheartj/ehae162 (PMC11089334; doi:10.1093/eurheartj/ehae162)
Supplement: ehae162_Supplementary_Data [file ehae162_supplementary_data.zip › 5_Supplementary Table2_v2.docx]

**Supplementary Table 2.** Unadjusted linear regression analysis of the overall study population exploring the association between baseline/clinical parameters and SNA (lAmygA/vmPFC). Standardized β coefficients and 95% confidence interval (CI) are presented. ^18^F-FDG-PET, 2-[^18^F]fluoro-2-deoxy-D-glucose positron emission tomography; BMI, body mass index; CAD, coronary artery disease; LV, left ventricular; LVEF, left ventricular ejection fraction; CRP, C-reactive protein; WBC, white blood cell count; NT-proBNP; N-terminal pro-brain natriuretic peptide

| **Factors** | **Standardized Coefficient (95%CI)** | **p-value** |
| --- | --- | --- |
| Age (years) | 0.298 (0.244, 0.352) | <0.001 |
| Sex (Male vs. Female) | 0.005 (-0.059, 0.068) | 0.881 |
| BMI (kg/m^2^) | 0.063 (-0.008, 0.135) | 0.081 |
| Heart rate (x10bpm) | 0.059 (-0.006, 0.125) | 0.077 |
| **Socioeconomic variables** |  |  |
| Living alone vs living not alone | -0.014 (-0.079, 0.051) | 0.681 |
| Low occupation skill level | 0.028 (-0.037, 0.093) | 0.396 |
| **Comorbidities** |  |  |
| Known comorbidities – Non cardiac | 0.058 (-0.011, 0.127) | 0.098 |
| Comorbidities – Cardiac | 0.058 (-0.011, 0.127) | 0.098 |
| History of cancer | -0.109 (-0.167, -0.052) | <0.001 |
| Chronic inflammatory disease | 0.054 (0.003, 0.104) | 0.038 |
| **Cardiovascular risk factors** |  |  |
| Obesity | 0.058 (-0.016, 0.132) | 0.125 |
| Diabetes | 0.143 (0.081, 0.205) | <0.001 |
| Dyslipidaemia | 0.102 (0.038, 0.166) | 0.002 |
| Hypertension | 0.181 (0.121, 0.240) | <0.001 |
| Family history of CAD | -0.027 (-0.085, 0.031) | 0.369 |
| Smoking | 0.023 (-0.040, 0.087) | 0.474 |
| **Medication** |  |  |
| Blood pressure/heart failure | 0.153 (0.092, 0.215) | <0.001 |
| Antiplatelet/anticoagulants | 0.121 (0.062, 0.179) | <0.001 |
| Anti-inflammatory drugs | 0.058 (-0.003, 0.119) | 0.060 |
| Antiarrhythmics | 0.061 (-0.009, 0.132) | 0.089 |
| Antidepressants | 0.060 (-0.003, 0.122) | 0.062 |
| Antidiabetic medication | 0.100 (0.038, 0.162) | 0.002 |
| Statins | 0.093 (0.033, 0.152) | 0.002 |
| **Echocardiography/MRI findings** |  |  |
| LV hypertrophy | 0.038 (-0.032, 0.109) | 0.289 |
| LVEF (%) | -0.009 (-0.076, 0.059) | 0.800 |
| LV wall motion abnormalities | 0.049 (-0.009, 0.106) | 0.096 |
| LV diastolic dysfunction | 0.103 (0.042, 0.164) | 0.001 |
| Valvular heart disease | 0.091 (0.031, 0.152) | 0.003 |
| **Inflammation markers (within 6 days of ^18^F-FDG-PET)** |  |  |
| CRP (mg/L) | 0.282 (0.186, 0.379) | <0.001 |
| WBC – Neutrophils (1000/microL) | 0.156 (0.044, 0.268) | 0.006 |
| WBC – Lymphocytes (1000/microL) | -0.006 (-0.015, 0.003) | 0.185 |
| **Inflammation markers (within 12 months of ^18^F-FDG-PET)** |  |  |
| CRP (mg/L) | 0.085 (0.018, 0.152) | 0.013 |
| WBC – Neutrophils (1000/microL) | -0.021 (-0.104, 0.062) | 0.621 |
| WBC – Lymphocytes (1000/microL) | -0.018 (-0.065, 0.028) | 0.444 |
| **Laboratory values (within 12 months of ^18^F-FDG-PET)** |  |  |
| Creatinine (umol/L) | 0.155 (0.092, 0.219) | <0.001 |
| NT-proBNP (ng/L) x10000 | 0.139 (0.069, 0.210) | <0.001 |
| Non-fasting glucose (mmol/L) | 0.108 (-0.003, 0.219) | 0.057 |
| **^18^F-FDG-PET/CT imaging parameters** |  |  |
| ^18^F-FDG uptake bone marrow (SUVmax) | 0.081 (0.021, 0.142) | 0.008 |
