# Supplementary material for: Imaging of the brain–heart axis: prognostic value in a European setting
Source: Eur Heart J. 2024 Apr 10;45(18):1613–30. doi: 10.1093/eurheartj/ehae162 (PMC11089334; doi:10.1093/eurheartj/ehae162)
Supplement: ehae162_Supplementary_Data [file ehae162_supplementary_data.zip › 5_Supplementary Table3_New.docx]

**Supplementary Table 3:** Comparative showing associations between stress-related neural activity (SNA) normalized to the ventromedial prefrontal cortex (lAmygA/vmPFC) or SNA normalized to background brain activity in the temporal lobe (lAmygA/temp) and outcomes: multivariable analysis with adjustment for various confounding factors. BMI, body-mass-index; CVRF, cardiovascular risk factor; HR, hazard ratio; lAmygdA, left amygdala metabolic activity; MACE, major adverse cardiovascular events; MRI, magnetic resonance imaging; SHR, sub-distribution hazard ratio;

|  | **MACE** | | | | **All-cause mortality** | | | |
| --- | --- | --- | --- | --- | --- | --- | --- | --- |
|  | **lAmygA/vmPFC** | | **lAmygA/temp** | | **lAmygA/vmPFC** | | **lAmygA/temp** | |
|  | **SHR (95%CI)** | **p-value** | **SHR (95%CI)** | **p-value** | **HR (95%CI)** | **p-value** | **HR (95%CI)** | **p-value** |
| **Unadjusted Model** |  |  |  |  |  |  |  |  |
| SNA per unit change | 8.37 (1.35, 51.96) | 0.023 | 14.60 (2.13, 100.10) | 0.006 | 26.40 (8.05, 86.59) | <0.001 | 98.56 (29.69, 327.22) | <0.001 |
| SNA high vs low* | 1.52 (1.05, 2.19) | 0.026 | 1.55 (1.04, 2.31) | 0.033 | 2.49 (1.96, 3.17) | <0.001 | 2.49 (1.86, 3.33) | <0.001 |
| **Model 1: Age, sex, BMI, heart rate** |  |  |  |  |  |  |  |  |
| SNA per unit change | 3.00 (0.26, 34.79) | 0.381 | 4.57 (0.34, 60.69) | 0.250 | 7.48 (1.51, 37.19) | 0.014 | 11.48 (2.29, 57.69) | 0.003 |
| SNA high vs low* | 1.41 (0.92, 2.17) | 0.118 | 1.18 (0.72, 1.95) | 0.516 | 1.95 (1.46, 2.60) | <0.001 | 1.73 (1.23, 2.45) | 0.002 |
| **Model 2: Age, sex, CVRFs, cardiac and non-cardiac comorbidities, sociocultural variables** |  |  |  |  |  |  |  |  |
| SNA per unit change | 2.33 (0.18, 30.20) | 0.517 | 2.92 (0.21, 41.41) | 0.429 | 4.57 (0.85, 24.54) | 0.076 | 6.73 (1.35, 33.66) | 0.020 |
| SNA high vs low* | 1.28 (0.84, 1.97) | 0.250 | 1.15 (0.68, 1.95) | 0.603 | 1.83 (1.37, 2.45) | <0.001 | 1.63 (1.15, 2.31) | 0.006 |
| **Model 3: Age, sex, and medication** |  |  |  |  |  |  |  |  |
| SNA per unit change | 2.44 (0.33, 18.31) | 0.385 | 2.85 (0.37, 22.23) | 0.317 | 6.14 (1.67, 22.50) | 0.006 | 14.90 (3.92, 56.56) | <0.001 |
| SNA high vs low* | 1.19 (0.81, 1.76) | 0.376 | 1.16 (0.76, 1.78) | 0.486 | 1.89 (1.47, 2.42) | <0.001 | 1.79 (1.33, 2.43) | <0.001 |
| **Model 4: Age, sex, and laboratory parameters** |  |  |  |  |  |  |  |  |
| SNA per unit change | 1.52 (0.15, 15.05) | 0.722 | 1.29 (0.11, 14.61) | 0.838 | 3.74 (0.88, 15.90) | 0.074 | 9.03 (1.99, 40.99) | 0.004 |
| SNA high vs low* | 1.33 (0.85, 2.07) | 0.207 | 0.93 (0.58, 1.48) | 0.751 | 1.78 (1.33, 2.40) | <0.001 | 1.52 (1.07, 2.16) | 0.020 |
| **Model 5: Age, sex, and cardiac imaging findings (echocardiography)** |  |  |  |  |  |  |  |  |
| SNA per unit change | 1.95 (0.15, 25.83) | 0.612 | 2.36 (0.21, 26.96) | 0.489 | 7.97 (1.55, 40.90) | 0.013 | 18.72 (3.71, 94.52) | <0.001 |
| SNA high vs low* | 1.18 (0.77, 1.82) | 0.445 | 1.13 (0.69, 1.87) | 0.621 | 1.83 (1.37, 2.46) | <0.001 | 1.77 (1.24, 2.55) | 0.002 |

*Cut-point defined by classification and regression tree analysis (CART) with high lAmygA/temp ≥ 0.735 and high lAmygA/vmPFC ≥ 0.727.
